# India should invest in the expansion of genomic epidemiology for vector-borne diseases filariasis, malaria and visceral leishmaniasis that are targeted for elimination

**DOI:** 10.1016/j.ijregi.2024.100453

**Published:** 2024-09-18

**Authors:** Nandini Singh, Amit Sharma

**Affiliations:** Molecular Medicine Group, International Centre for Genetic Engineering and Biotechnology, New Delhi, India

**Keywords:** Filariasis, Genomic epidemiology, Infectious diseases, Influenza, Malaria, Visceral leishmaniasis

## Abstract

Genomic epidemiology (GE) is an integration of genomics and epidemiology. The field has evolved significantly in the past decade, enhancing our understanding of genetic susceptibility, drug resistance, disease transmission patterns, outbreak surveillance, and vaccine development. It employs an arsenal of advanced tools such as whole-genome sequencing and single-nucleotide polymorphisms for analysis, tracing pathogen evolution, mapping genetic variations, and tracking drug resistance. The role of GE in infectious disease management extends beyond outbreak control to routine public health practices, precision medicine, and determining treatment policies. The expansion of GE can significantly bolster global health defenses by effectively enabling the detection and response to emerging health threats. However, challenges such as sampling bias, data quality, integration, standardization of computational pipelines, and need for trained personnel remain. To tackle these challenges, we must invest in building capacity, improving infrastructure, providing training, and fostering collaborations between scientists and public health officials. Concerted efforts must focus on overcoming existing hurdles and promoting seamless integration of basic research into public health frameworks to fully realize its potential. It is timely for India to rapidly expand its base in GE to gain valuable insights into genetic variations and disease susceptibilities. This will provide a fillip towards eliminating the three dominant vector-borne diseases in India: filariasis, malaria, and visceral leishmaniasis.

## Highlights

• Genomic epidemiology (GE) is the integration of genomics and epidemiology.
• GE utilizes an arsenal of advanced tools such as whole-genome sequencing and single-nucleotide polymorphisms for analysis.
• Some of the key challenges include sampling bias, data quality, integration, etc.
• It is an opportune moment for India to enhance GE for diseases.
• We should utilize GE in neglected tropical diseases to improve public health responses.

## Introduction

Genomic epidemiology (GE) has its roots in molecular epidemiology. It encompasses genomics, the science of genes and their functions, and epidemiology, the science of disease patterns and their determinants. It relies heavily on biostatistics, genetics, molecular epidemiology, nucleic acid sequencing, proteomics and transcriptomics. These tools can address fundamental inquiries regarding susceptibility and causal genomic variations [1]. GE gained worldwide attention during the COVID-19 pandemic, where it drove the identification of strains of the virus, allowed tracking of virus spread, followed viral evolution, and contributed towards the development of vaccines.

Beyond fingerprinting pathogens, tracking drug resistance, unveiling evolutionary links, enhancing disease surveillance, and contributing to therapeutic development, it is also utilized in precision medicine. It provides insights into the genetics of the individuals and the population, facilitating the development of tailored interventions [2]. The genomic analysis in one health approach takes a holistic view by integrating human, pathogen, and environmental data, which can predict and prevent zoonotic outbreaks, thus strengthening pandemic preparedness [3]. A study conducted in Canada at a tertiary-care hospital utilized localized, on-demand genomic sequencing of SARS-CoV-2 in real-time to identify distinct transmission clusters and modes of transmission, leading to enhanced control measures to contain the infection, which could help prevent future nosocomial outbreaks [4]. GE optimizes treatment strategies by tracking antimicrobial resistance, identifying causal organisms through phylogenetic analysis, predicting infection outbreaks, and informing infection preventive strategies [5,6]. Phylogenomic analysis can aid in drug-discovery studies by revealing the origin of genes and their relatedness in prokaryotes vs eukaryotes, as exemplified by the analysis of methionyl-tRNA synthetase in *Plasmodium falciparum* (PfMRS) [7]. Precise DNA sequencing of pathogens at high resolution and modeling advancements have enabled quick monitoring, coordination, and resource allocation for emerging outbreaks [8]. Influenza A virus, with its frequently mutating segmented RNA genome, poses a persistent health threat. By tracking recombination in genes for glycoproteins hemagglutinin (HA) and neuraminidase (NA), annual adjustments to seasonal flu vaccines are possible, helping to mitigate inter-seasonal variability and guiding vaccine development [9,10]. Large datasets of GE are also utilized to characterize novel pathways for acquiring resistance due to human interference, as also shown for *Streptococcus pneumoniae* [11].

The diverse arsenal of tools extensively employed in GE include methods to unravel information from genetics to the genomics level, followed by clinically relevant inferences and their implications from an individual to the population level. Whole-genome sequencing reveals the genetic makeup of hosts and pathogens, offering extensive data for in-depth analysis. It is being extensively used globally in infection prevention and preparedness efforts [12]. Single-nucleotide polymorphism analysis can identify specific points of variation in pathogen genomes, which helps delineate the different strains and their transmission patterns [5,6]. Phylogenomics can recreate the evolutionary history of pathogens to understand their origins, dissemination, and potential for future evolution. Phylodynamic methodologies integrate evolutionary, demographic, and epidemiologic principles, aiding in monitoring genetic alterations in viruses, detecting emerging variants, and formulating public health strategies [13].

The importance of GE is highlighted in regions with diverse epidemiologic profiles where routine surveillance every 1-2 years is essential because pathogens constantly develop new variants. In some cases, when the selective pressure of specific drugs is lifted, pathogens can revert to their wild-type genome. This reversion can only be detected through regular pathogen surveillance. In addition, the emergence of drug resistance is common in many parasites, and it can diminish the efficacy of current treatments. These are routinely tracked with GE.

## Notable examples of GE in infectious diseases

### SARS-CoV-2

GE played a critical role in the COVID-19 pandemic locally and globally. GE informed decisions and policy changes via tracking of SARS-CoV-2 [14]. The diagnosis of COVID-19 became possible due to the availability of the genomic sequence of the virus, which was utilized for the laboratory detection of cases using real-time reverse transcriptase-polymerase chain reaction [15]. Early advances were based on the Wuhan isolate's reference sequence, but gradually, new variants arose. Point mutations were important tracking targets as they can confer resistance to neutralizing antibodies, making existing treatment options ineffective [16]. Some mutations, for example, E484, chiefly in the receptor-binding domain of spike (S) protein, allowed the virus to evade the immune response and recognition by polyclonal antibodies [17]. The S protein of SARS-CoV-2 was intensely characterized because of its multifaceted roles, such as recognition of target, cellular entry, and endosomal escape [18]. It remains a hotspot of mutations in several variants of the virus. With time, some of the mutations in receptor-binding domain of S protein, such as D614G (G614 variant), N501Y in Alpha (B.1.1.7) and Beta (B.1.351), E484K in Beta and Gamma (P.1), K417N/T in Beta and Gamma, and L452R in Delta and Epsilon (B.1.427/B.1.429) became signature mutations [19].

The continuous surveillance of mutations and emerging variants through various platforms, such as World Health Organization (WHO)’s COVID dashboard and Outbreak.info, and the availability of genomic sequence data at GISAID (the Global Initiative on Sharing All Influenza Data) along with Nextstrain allowed researchers to be in sync with the evolving SARS-CoV-2 virus [16,20]. Regular genomic surveillance informed transmission dynamics in populations at global and local levels. This allowed tailored public health interventions such as targeted testing, contact tracing, quarantine measures, and variable vaccination strategies [21]. Vaccine efficacy studies and tracking of phenotypic changes due to mutations in S protein, which could change the transmissibility, binding affinity, and antigenicity, were also performed routinely [22]. Thus, GE proved to be an indispensable tool during the COVID-19 pandemic and paved the way for better containment strategies for future outbreaks.

### Influenza

The influenza virus poses a persistent and unpredictable global health threat, marked by recurring pandemics, with the most recent four occurring within a century, i.e., the Spanish flu in 1918 (H1N1), the Asian flu in 1957 (H2N2), the Hong Kong flu in 1968 (H3N2), and the swine-origin flu in 2009 (H1N1/09) [10]. Influenza viruses have a segmented RNA genome that codes for envelope glycoproteins HA and NA, as well as several other proteins. Influenza A viruses affect various animals, whereas influenza B viruses are found in humans only. Avian influenza A viruses, based on the surface glycoproteins, are classified into different subtypes. Up to 18 HA and 11 NA subtypes have been identified to date. Whole-genome sequencing enabled mutational tracking in these glycoproteins. It facilitated the epidemiologic surveillance of inter- and intra-seasonal variability and the genetic background of viruses for phylogenetic analysis [10]. In the 1950s, the WHO initiated a comprehensive global surveillance network for influenza, spanning institutions across 122 member states. These surveillance mechanisms actively monitor influenza strains circulating among human and animal populations, enabling the prompt identification of strains with pandemic potential, which is analyzed via computational models for potential impact on the population [9]. Given the frequent emergence of new strains, adjustments to the composition of the seasonal flu vaccine are made annually, guided by assessments of viral mutations and transmission dynamics. Consequently, vaccines are tailored to target three or four strains anticipated to be prevalent in the upcoming flu season. Different reassorting strains with recombination in genes for HA and NA of H1N1 clade, and reduced vaccine efficacy due to changes in antigen characteristics of reassortant strains of H3N2 were both tracked using GE [9]. Therefore, GE plays a crucial role by providing insights into influenza viruses' genetic makeup and evolutionary trajectories, informing vaccine development strategies, and enhancing global preparedness against outbreaks [23].

### Ebola virus outbreaks

Similarly, the recent series of Ebola outbreaks spanning from 2014 to 2016 in West Africa and from 2018 to 2020 in the eastern Democratic Republic of Congo resulted in widespread morbidity and mortality. Four lethal infection-causing strains of the Ebola virus (EBOV) have been identified, namely Tai Forest, Bundibugyo, Sudan, and Zaire (EBOV) [24]. Historically, EBOV has majorly affected endemic regions and only a relatively small number of people globally. The recent outbreaks, however, have demonstrated transmission to nonendemic regions, prompting the scientific community to hasten control measures and develop vaccines. Among the most advanced vaccines in the US and Europe are Ervebo (rVSV-EBOV), Zabdeno/Mvabea (Ad26-ZEBOV/MVA-BN-Filo), and cAd3-EBOZ [24]. GE has been significant in tracking mutations in glycoprotein 1,2 which can decrease vaccine effectiveness [24]. GE can also target vaccine development by covering outbreak-level events to epidemic-level trends, revealing the outbreak's source, identifying multiple transmission chains, and aiding containment efforts. Substantial advancements were achieved in the fight against Ebola due to a blend of DNA sequencing technologies, internationally endorsed containment strategies, widespread distribution of crucial information to the public, modern technology for improved outbreak surveillance, enhanced implementation of active surveillance, targeted travel limitations, and quarantine measures [25].

### Zika virus outbreaks

Zika virus (ZIKV) outbreaks in 2015-2016 led the WHO to declare it as a Public Health Emergency of International Concern (PHEIC) in response to the correlation between Zika virus, microcephaly and other neurologic disorders. According to WHO, cases were also reported from Indian states such as Kerala, Maharashtra, Uttar Pradesh, Gujarat, Rajasthan and Madhya Pradesh during 2018-2021. Given that ZIKV can also be transmitted through sexual contact, approximately 1% of infections in the USA and Europe during 2015-2017 were reported to have been acquired via this route [26]. GE has helped trace viral mutations such as S139N, understand its migration patterns, and inform public health systems. Various vaccines are under clinical trials and WHO is actively engaged in genomic surveillance of the virus. According to the serologic and genomic studies, there is a single serotype of the virus, which is divided into three distinct genetic lineages: East African (including the first isolate from Uganda, MR766), West African, and Asian (which includes the first isolate from Asia, P6-740, along with all current strains from Asia, Oceania, and the US) [27]. Combining immunoinformatics and GE is a powerful approach for ZIKV research. The integration of genomic data with immune response modeling can help in vaccine development, personalized medicine, and in predictive modeling for the effectiveness of interventions [27].

## GE of filariasis, malaria, and visceral leishmaniasis

Being home to a very diverse population, India inevitably faces a complex variety of infectious diseases. The importance of GE is not limited to pandemics only. The three major vector-borne diseases targeted for elimination in India over the next few years are filariasis, malaria, and visceral leishmaniasis (VL). It is an opportune time to invest significantly in the GE of these three pathogens to understand and inform public health interventions required for their elimination from India.

Filariasis, or lymphatic filariasis (LF), is a neglected tropical disease transmitted by mosquito species such as *Culex, Anopheles* and *Aedes* in different regions worldwide. This disease is caused by infectious nematode (roundworm) parasites, specifically *Wuchereria bancrofti, Brugia malayi* and *Brugia timori*, which inhabit the lymphatic vessels. Data from WHO suggests that *W. bancrofti* accounts for ∼90% of the cases. Infected individuals suffer from symptoms like lymphedema, elephantiasis, and scrotal swelling. According to the WHO, over 882 million people in 44 countries are at risk of LF as of 2023. The Mass Drug Administration (MDA) program under the Global Program to Eliminate Lymphatic Filariasis (GPELF) framework of the WHO was launched in 2000, which has helped decrease the number of countries that require preventive chemotherapy to 44. In India, the National Center for Vector Borne Diseases Control (NCVBDC) oversees a comprehensive initiative to prevent and control vector-borne diseases such as LF, lala-azar, malaria, Japanese encephalitis, dengue, and chikungunya. It aims explicitly to eliminate LF, kala-azar, and malaria in the coming years. According to the NCVBDC report 2023, there are 20 states with LF endemicity, with 333 districts reporting endemic status. In addition, ∼90% of the LF burden is contributed by eight states: Uttar Pradesh, Bihar, Jharkhand, West Bengal, Chhattisgarh, Maharashtra, Odisha, and Madhya Pradesh. GE can be valuable for xenomonitoring, and for studying populations by analyzing whole-genome variations within and between parasite populations, which can help in understanding drug resistance, disease transmission patterns, and therapeutic efficacy. It has proven helpful in surveillance efforts as demonstrated by identification of Wb123 as a marker for early *W. bancrofti* infection, which was subsequently utilized for monitoring MDA [28]. The insights gained can be used to determine optimal treatment cessation, assess disease recurrence risks from infected individuals or vectors, and evaluate the long-term viability of the MDA program, especially if poor drug response is hereditary [29]. The limited number of anti-filarial drugs call for the drug-discovery process to be hastened, which, at its core, will also need GE. It can also aid in understanding geographic and temporal differences in drug response. The database https://parasite.wormbase.org/index.html has all the annotated genomic sequences of these parasites, and India can initiate multi-state genomic surveillance of the parasites to distill the benefits of GE in disease management [29].

Malaria is an infectious disease spread by mosquitoes and caused by parasites of the *Plasmodium* genus. Of the five species that frequently infect humans, *P. falciparum* and *Plasmodium vivax* are noted for their severe symptoms. It remains a global health challenge, particularly in tropical and sub-tropical regions. According to the WHO, in 2022, worldwide, there were ∼249 million reported cases of malaria and ∼608,000 deaths across 85 countries. Traditional epidemiological methods to estimate and contain malaria through National Malaria Control Programmes (NMCPs) were sufficient earlier, but now more detailed interventions are required. GE has revolutionized our understanding of malaria by emerging as a powerful tool for tracking parasite diversity, transmission dynamics, drug resistance, and population structure of parasites [30]. The Malaria Genomic Epidemiology Network (MalariaGEN) offers a global platform for facilitating the generation, integration, and dissemination of genetic and genomic data related to malaria parasites and vectors. Over time, the malaria parasite evolves naturally or under the selective pressure of drugs, and new mutations arise, decreasing antimalarial drug's efficacy. Genomic surveillance of *Plasmodium* parasites has identified mutations in genes encoding dihydropteroate synthase and dihydrofolate reductase enzymes, which confer resistance to sulfadoxine-pyrimethamine [31]. Gene deletions in the histidine-rich protein-2 (hrp2/3) gene of *P. falciparum* lead to false negatives when using a rapid diagnostic test, thus influencing the actual number of infections [32]. Similarly, the *P. falciparum* multidrug resistance 1 (Pf MDR1) gene is linked to resistance against several anti-malaria therapies, whereas mutations in *P. falciparum* Kelch-13 (Pfk13) and *P. falciparum* Coronin (Pf Coronin) genes can confer partial artemisinin resistance [[33], [34], [35]]. The vaccine development process has been hastened by GE as the latter plays a crucial role in identifying markers associated with protective immunity against malaria [36]. Study of genome sequences has enabled researchers to identify antigenic targets for vaccine development and assess the impact of genetic variation on vaccine efficacy [36]. GE has identified genetic variations in the *P. falciparum* circumsporozoite protein, apical membrane antigen 1, and thrombospondin-related adhesion protein, which are vaccine candidates for malaria [37]. These variations impact the antigenic characteristics and immune responses to these proteins by host's immune system. Insights from these studies have influenced the advancement of malaria vaccines, focusing on conserved sequences of the parasite genome to address challenges posed by antigenic diversity and mutations that allow the parasite to evade immune recognition [38]. Regular surveillance of parasite resistance against drugs and vector resistance to insecticides is also crucial, as reported recently from 15 states of India [39].

The dried blood spot sample collection method is advantageous for routine GE. Dried blot spots are the most accessible to collect in settings with minimal healthcare facilities, and the downstream procedures for handling and testing are easy [40].

VL, also known as kala-azar, is the most severe and fatal form (if untreated) of leishmaniasis. It is caused by protozoan parasites of the *Leishmania* genus and is transmitted by sandflies of the genus *Phelebotomus.* The reticuloendothelial system is majorly affected and abundant parasites persist in the liver and spleen. According to the WHO, 99 countries were endemic to leishmaniasis in 2022, out of which 71 showed endemicity to visceral and cutaneous leishmaniasis, nine to VL only and 19 to cutaneous leishmaniasis only. In addition, 85% of the global VL cases were reported from seven countries, including India. According to the NCVBDC, in India, *Leishmania donovani* is the only causative agent of this disease, showing endemicity in eastern states, namely Bihar, Jharkhand, Uttar Pradesh, and West Bengal. It is estimated that 165 million people of these four states are at risk. GE has helped track the genetic diversity within the *Leishmania* genus. It has also illuminated variations in specific genes, such as *L. donovani* aquaglyceroporin 1. This gene is involved in the metabolism of drugs and contributes to resistance against antimonial treatments by preventing the transport of trivalent antimonials. The propagation of this resistance in response to drug treatment through genetic recombination within populations has also been documented in a study from the Indian subcontinent [41]. Regular genomic surveillance of parasite populations is essential for identifying and addressing instances of treatment failure that may arise due to mutations [41]. Preliminary detection of VL can be achieved by targeting kinetoplast DNA through polymerase chain reaction assays [42]. Preclinical studies of a recombinant protein-based vaccine candidate, LEISH-F3+GLA-SE, have shown encouraging results [43]. The *Leishmania* genus, along with its vector, exhibits substantial genetic diversity. Understanding their genomics is crucial for effective parasite and vector control strategies.

**Figure 1:**
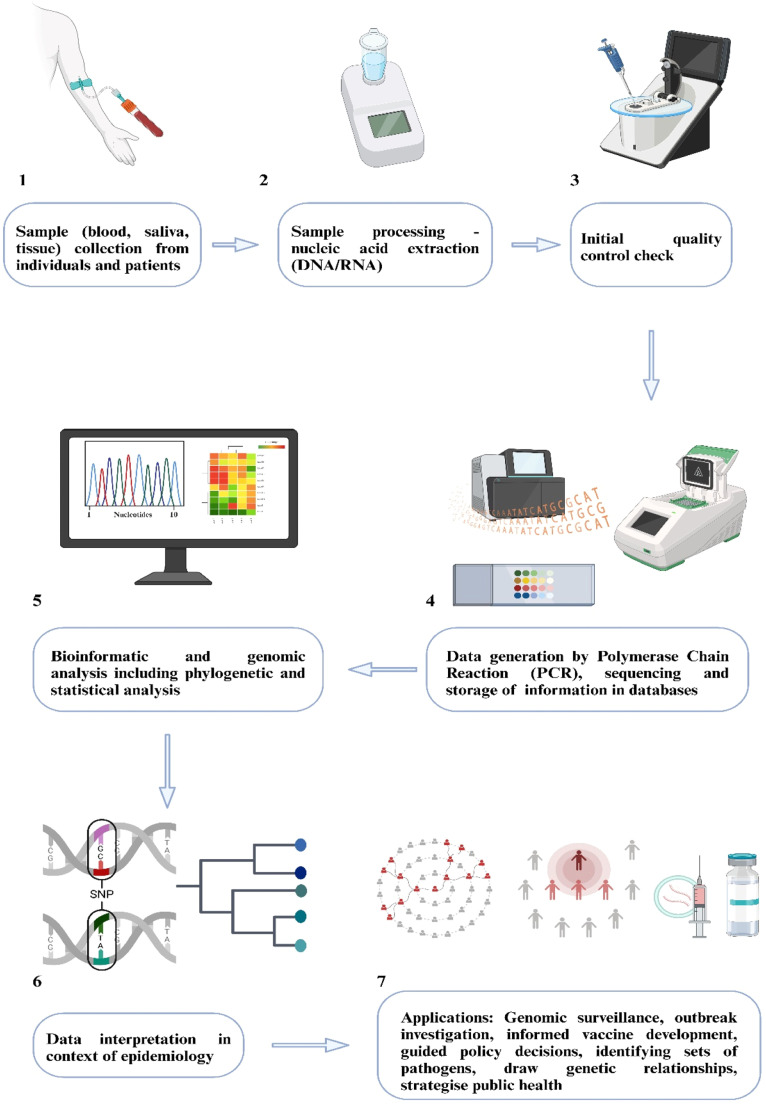
Summarizes the typical genomic epidemiology workflow, from sample collection to different applications. Created using Biorender.com. SNP: Single Nucleotide Polymorphism.

Figure 1 summarizes the typical GE workflow, from sample collection to different applications. Recent analysis has delineated co-endemic and congruent regions in India where these three diseases intersect. Data from 2016 to 2019 reveal that all three diseases afflict ∼25 districts, whereas ∼197 districts are impacted by any two [44]. Beyond overlapping geographic locales, these diseases share epidemiological characteristics. These include environmental, socioeconomic, demographic, cultural and health-seeking features characteristics of at-risk populations. An integrated genomic surveillance approach covering both epidemiological and entomological facets, can contribute towards elimination of these diseases [44].

## Limitations of genomic epidemiology

Although GE has progressed since its introduction, it still has pitfalls. To fully harness its potential, several hurdles need to be overcome. The efficiency of genomic surveillance varies with the method of sample collection, correct case identification, use of the proper diagnostic test, pathogen load, and the time gap from case identification to genome sequencing [1]. Sampling bias impacts the phylogeographic analysis by over-representing the data, which can also be attributed to the limited access to sequencing technologies and bioinformatics expertise in technologically challenged regions. Chief concerns are integration of genomic data with metadata from different origins, development of efficient computational algorithms to handle extensive datasets, and establishment of sampling frameworks to ensure reliable findings [[45], [46], [47]]. The standardization of bioinformatics pipelines is yet another challenge in this field, and the lack of it can increase error rates in the data. Compartmental collection of clinical and metadata serves as a setback for their linking [46] and should be avoided in GE. Inconsistent data collection and lack of sharing of clinical and demographic data can hinder the compilation of comprehensive datasets, leading to challenges in data reporting and limiting timely access to information. Delays in analysis of genomic surveillance data can alter outbreak mitigation strategies. The costs of genomic sequencing, ethical and legal concerns, social implications, and data privacy must be addressed [46]. To tackle these hurdles, sustained investment in capacity building, technology transfer, standardization, and infrastructure is required. The collaboration among public health system, scientists and policymakers can help realize the full potential of GE [2].

## Conclusion

GE is a transformative force with the potential to reshape public health by improving disease management. During the SARS-CoV-2 pandemic, it was critical in tracking virus mutations and in guiding global response efforts. Similarly replicated efforts are needed for every infectious disease. As India is targeting the elimination of its three major vector-borne parasite diseases, it can consider enhancing investment into parasite and vector genomic sequencing as that will provide a bioinformatics platform to harness the benefits of GE. Besides funds, this will require a seamless collaboration between stakeholders such as basic scientists, public health managers, and policymakers. It is an opportune moment to initiate a comprehensive program for integrating genomic data with field epidemiology in context of filaria, malaria and leishmaniasis in India.

## Declarations of competing interest

The authors have no competing interests to declare.

## References

[1] Duggal P, Ladd-Acosta C, Ray D, Beaty TH. (2019). The evolving field of genetic epidemiology: from familial aggregation to genomic sequencing. Am J Epidemiol.

[2] Khoury MJ, Holt KE. (2021). The impact of genomics on precision public health: beyond the pandemic. Genome Med.

[3] Erkyihun GA, Alemayehu MB. (2022). One health approach for the control of zoonotic diseases. Zoonoses.

[4] Benoit P (2023). On-demand, hospital-based, severe acute respiratory coronavirus virus 2 (SARS-CoV-2) genomic epidemiology to support nosocomial outbreak investigations: a prospective molecular epidemiology study. Antimicrob Steward Healthc Epidemiol.

[5] Shastry BS. (2007). SNPs in disease gene mapping, medicinal drug development and evolution. J Hum Genet.

[6] Vankadari N. (2020). Overwhelming mutations or SNPs of SARS-CoV-2: A point of caution. Gene.

[7] Hussain T, Yogavel M, Sharma A. (2015). Inhibition of protein synthesis and malaria parasite development by drug targeting of methionyl-tRNA synthetases. Antimicrob Agents Chemother.

[8] Stockdale JE, Liu P, Colijn C. (2022). The potential of genomics for infectious disease forecasting. Nat Microbiol.

[9] Maljkovic Berry I, Melendrez MC, Li T, Hawksworth AW, Brice GT, Blair PJ (2016). Frequency of influenza H3N2 intra-subtype reassortment: attributes and implications of reassortant spread. BMC Biol.

[10] Becker T, Elbahesh H, Reperant LA, Rimmelzwaan GF, Osterhaus ADME. (2021). Influenza vaccines: successes and continuing challenges. J Infect Dis.

[11] Croucher NJ, Harris SR, Fraser C, Quail MA, Burton J, Van Der Linden M (2011). Rapid pneumococcal evolution in response to clinical interventions. Science.

[12] Doll M, Bryson AL, Palmore TN. (2024). Whole genome sequencing applications in hospital epidemiology and infection prevention. Curr Infect Dis Rep.

[13] Attwood SW, Hill SC, Aanensen DM, Connor TR, Pybus OG. (2022). Phylogenetic and phylodynamic approaches to understanding and combating the early SARS-CoV-2 pandemic. Nat Rev Genet.

[14] Saravanan KA, Panigrahi M, Kumar H, Rajawat D, Nayak SS, Bhushan B (2022). Role of genomics in combating COVID-19 pandemic. Gene.

[15] Wang H, Li X, Li T, Zhang S, Wang L, Wu X (2020). The genetic sequence, origin, and diagnosis of SARS-CoV-2. Eur J Clin Microbiol Infect Dis.

[16] Kumar S, Chandele A, Sharma A. (2021). Current status of therapeutic monoclonal antibodies against SARS-CoV-2. PLoS Pathog.

[17] Greaney AJ, Loes AN, Crawford KH, Starr TN, Malone KD, Chu HY (2021). Comprehensive mapping of mutations in the SARS-CoV-2 receptor-binding domain that affect recognition by polyclonal human plasma antibodies. Cell Host Microbe.

[18] Huang Y, Yang C, Xu XF, Xu W, Liu SW. (2020). Structural and functional properties of SARS-CoV-2 spike protein: potential antivirus drug development for COVID-19. Acta Pharmacol Sin.

[19] Magazine N, Zhang T, Wu Y, McGee MC, Veggiani G, Huang W. (2022). Mutations and evolution of the SARS-CoV-2 spike protein. Viruses.

[20] Gangavarapu K, Latif AA, Mullen JL, Alkuzweny M, Hufbauer E, Tsueng G (2023). Outbreak.info. Outbreak.info genomic reports: scalable and dynamic surveillance of SARS-CoV-2 variants and mutations. Nat Methods.

[21] Novelli G, Biancolella M, Mehrian-Shai R, Colona VL, Brito AF, Grubaugh ND (2021). COVID-19 one year into the pandemic: from genetics and genomics to therapy, vaccination, and policy. Hum Genomics.

[22] Tosta S, Moreno K, Schuab G, Fonseca V, Segovia FM, Kashima S (2023). Global SARS-CoV-2 genomic surveillance: what we have learned (so far). Infect Genet Evol.

[23] Harrington WN, Kackos CM, Webby RJ. (2021). The evolution and future of influenza pandemic preparedness. Exp Mol Med.

[24] Woolsey C, Geisbert TW. (2021). Current state of Ebola virus vaccines: A snapshot. PLOS Pathog.

[25] Wojda TR, Valenza PL, Cornejo K, McGinley T, Galwankar SC, Kelkar D (2015). The Ebola outbreak of 2014–2015: from coordinated multilateral action to effective disease containment, vaccine development, and beyond. J Glob Infect Dis.

[26] Essink B, Chu L, Seger W, Barranco E, Le Cam N, Bennett H (2023). The safety and immunogenicity of two Zika virus mRNA vaccine candidates in healthy Flavivirus baseline seropositive and seronegative adults: the results of two randomised, placebo-controlled, dose-ranging, phase 1 clinical trials. Lancet Infect Dis.

[27] Pattnaik A, Sahoo BR, Pattnaik AK. (2020). Current status of Zika virus vaccines: successes and challenges. Vaccines.

[28] Bennuru S, O'Connell EM, Drame PM, Nutman TB (2018). Mining filarial genomes for diagnostic and therapeutic targets. Trends Parasitol.

[29] Hedtke SM, Kuesel AC, Crawford KE, Graves PM, Boussinesq M, Lau CL (2020). Genomic epidemiology in filarial nematodes: transforming the basis for elimination program decisions. Front Genet.

[30] Dalmat R, Naughton B, Kwan-Gett TS, Slyker J, Stuckey EM (2019). Use cases for genetic epidemiology in malaria elimination. Malar J.

[31] Chaturvedi R, Chhibber-Goel J, Verma I, Gopinathan S, Parvez S, Sharma A. (2021). Geographical spread and structural basis of sulfadoxine-pyrimethamine drug-resistant malaria parasites. Int J Parasitol.

[32] Mekonen B, Dugassa S, Feleke SM, Dufera B, Gidisa B, Adamu A (2024). Widespread pfhrp2/3 deletions and HRP2-based false-negative results in southern Ethiopia. Malar J.

[33] Chhibber-Goel J, Sharma A. (2019). Profiles of Kelch mutations in Plasmodium falciparum across South Asia and their implications for tracking drug resistance. Int J Parasitol Drugs Drug Resist.

[34] Kong X, Feng J, Xu Y, Yan G, Zhou S. (2022). Molecular surveillance of artemisinin resistance-related Pfk13 and pfcrt polymorphisms in imported Plasmodium falciparum isolates reported in eastern China from 2015 to 2019. Malar J.

[35] Ward KE, Fidock DA, Bridgford JL. (2022). Plasmodium falciparum resistance to artemisinin-based combination therapies. Curr Opin Microbiol.

[36] Su X, Stadler RV, Xu F, Wu J. (2023). Malaria genomics, vaccine development, and microbiome. Pathogens.

[37] Neafsey DE, Taylor AR, MacInnis BL. (2021). Advances and opportunities in malaria population genomics. Nat Rev Genet.

[38] Kale S, Pande V, Singh OP, Carlton JM, Mallick PK. (2021). Genetic diversity in two leading Plasmodium vivax malaria vaccine candidates AMA1 and MSP119 at three sites in India. PLoS Negl Trop Dis.

[39] Raghavendra K, Rahi M, Verma V, Velamuri PS, Kamaraju D, Baruah K (2022). Insecticide resistance status of malaria vectors in the malaria endemic states of India: implications and way forward for malaria elimination. Heliyon.

[40] Nain M, Sinha A, Sharma A. (2023). Dried blood spots: a robust tool for malaria surveillance in countries targeting elimination. J Vector Borne Dis.

[41] Imamura H, Downing T, Van den Broeck F, Sanders MJ, Rijal S, Sundar S (2016). Evolutionary genomics of epidemic visceral leishmaniasis in the Indian subcontinent. eLife.

[42] Abbasi I, Aramin S, Hailu A, Shiferaw W, Kassahun A, Belay S (2013). Evaluation of PCR procedures for detecting and quantifying Leishmania donovani DNA in large numbers of dried human blood samples from a visceral leishmaniasis focus in Northern Ethiopia. BMC Infect Dis.

[43] Dinc R. (2022). Leishmania vaccines: the current situation with its promising aspect for the future. Korean J Parasitol.

[44] Rahi M, Chaturvedi R, Das P, Sharma A. (2021). India can consider integration of three eliminable disease control programmes on malaria, lymphatic filariasis, and visceral leishmaniasis. PLOS Pathog.

[45] Rahi M, Sharma A. (2020). For malaria elimination India needs a platform for data integration. BMJ Glob Health.

[46] Ling-Hu T, Rios-Guzman E, Lorenzo-Redondo R, Ozer EA, Hultquist JF. (2022). Challenges and opportunities for global genomic surveillance strategies in the COVID-19 era. Viruses.

[47] Rahi M, Sharma A. (2022). Malaria control initiatives that have the potential to be gamechangers in India's quest for malaria elimination. Lancet Reg Health Southeast Asia.

